# Biomarkers for risk stratification and antibiotic stewardship in elderly patients

**DOI:** 10.1007/s40520-023-02388-w

**Published:** 2023-03-30

**Authors:** Marco Falcone, Michael Bauer, Ricard Ferrer, Gaëtan Gavazzi, Juan Gonzalez del Castillo, Alberto Pilotto, Philipp Schuetz

**Affiliations:** 1 Department of Infectious Diseases, Pisa University Hospital, Pisa, Italy; 2 Department of Anesthesiology and Intensive Care Medicine, Jena University Hospital, Jena, Germany; 3 Intensive Care Department, Hospital Universitari Vall d’Hebron, SODIR Research Group, Vall d’Hebron Institut de Recerca (VHIR), Barcelona, Spain; 4 Clinical Geriatrics Unit, Grenoble University Hospital, Grenoble, France; 5 Department of Emergency Medicine, Clínico San Carlos Hospital, IdISSC, Complutense University, Madrid, Spain; 6 Department of Interdisciplinary Medicine, University of Bari, Bari, Italy; 7 Department of Geriatric Care, OrthoGeriatrics and Rehabilitation, Galliera Hospital, Genoa, Italy; 8 Internal Medicine and Emergency Medicine, Aarau Hospital, Aarau, Switzerland

**Keywords:** Elderly, Geriatrics, Procalcitonin, Antibiotic stewardship, Infection

## Abstract

**Purpose:**

Optimal treatment of infections in the elderly patients population is challenging because clinical symptoms and signs may be less specific potentially resulting in both, over- and undertreatment. Elderly patients also have a less pronounced immune response to infection, which may influence kinetics of biomarkers of infection.

**Methods:**

Within a group of experts, we critically reviewed the current literature regarding biomarkers for risk stratification and antibiotic stewardship in elderly patients with emphasis on procalcitonin (PCT).

**Results:**

The expert group agreed that there is strong evidence that the elderly patient population is particularly vulnerable for infections and due to ambiguity of clinical signs and parameters in the elderly, there is considerable risk for undertreatment. At the same time, however, this group of patients is particularly vulnerable for off-target effects from antibiotic treatment and limiting the use of antibiotics is therefore important. The use of infection markers including PCT to guide individual treatment decisions has thus particular appeal in geriatric patients. For the elderly, there is evidence that PCT is a valuable biomarker for assessing the risk of septic complications and adverse outcomes, and helpful for guiding individual decisions for or against antibiotic treatment. There is need for additional educational efforts regarding the concept of “biomarker-guided antibiotic stewardship” for health care providers caring for elderly patients.

**Conclusion:**

Use of biomarkers, most notably PCT, has high potential to improve the antibiotic management of elderly patients with possible infection for improving both, undertreatment and overtreatment. Within this narrative review, we aim to provide evidence-based concepts for the safe and efficient use of PCT in elderly patients.

## Introduction

With the recent advances in medical care and as a result of demographic trends, the population of elderly and polymorbid patients is steadily growing. This population, however, is particularly vulnerable for systemic infections, which is responsible for the majority of deaths in this patient population [1, 2]. Early identification of infection and appropriate initial management including start of antibiotic treatment and fluid resuscitation is the first crucial step to improve clinical outcomes [2, 3]. Additionally, once treated, monitoring of patients during treatment may allow timely escalation of therapy in case of treatment failure and de-escalation in case of a favourable treatment response [2]. Importantly, this population of patients is also highly vulnerable regarding off-target effects from antibiotic treatments and thus early de-escalation or cessation of antibiotic treatment, once the condition has stabilized, is mandatory.

In clinical practice, the decision for or against antibiotic treatment based exclusively on clinical grounds has many drawbacks due to limited sensitivity and specificity of clinical signs and symptom [4]. This is particularly true in the elderly population, where the clinical presentation of an infection is less pronounced compared to younger patients. Herein, the use of specific blood biomarkers mirroring specific physio-pathological pathways may help to better estimate the likelihood and the resolution of infection, which in turn potentially improves clinical decision-making [5–7]. Among different blood markers, serum procalcitonin (PCT) has emerged as a host-derived biomarker that increases in bacterial infections and furthermore provides prognostic information, and thus may improve sepsis management [8–10]. The kinetics of PCT in an infected patient provide information about the recovery and risk for adverse outcome, which in turn may influence decisions about the duration of antibiotic treatment [8, 9, 11]. Multiple randomized controlled trials (RCT) have investigated the benefits of using serum PCT levels to guide whether and for how long antibiotic therapy is used—a process referred to as PCT-guided antibiotic stewardship. Such interventional studies were done in patients with different types of infections including those with sepsis hospitalized in the intensive care unit (ICU) [1, 12–22]. There are several trials and meta-analyses from such trials suggesting that PCT use decreases antibiotic exposure with beneficial effects on clinical outcomes including lower mortality in patients with respiratory tract infections, sepsis and blood stream infection [23–27]. As a limitation, only few trials have specifically targeted the elderly patient population and it remains somewhat unclear how well results from trials focusing on younger patients can be transferred to the elderly patient population. This is particularly true in regard to Immunosenescence, a process of immune dysfunction that occurs with age and includes remodeling of lymphoid organs, leading to changes in the immune function of the elderly [28]. Older patients thus may present with a less pronounced immune response, specifically regarding cytokine and biomarker responses to infection compared to younger ones [28, 29]. Immunosenescence reflects a facet of human aging and has been associated with a decline in adaptive and innate immunity [28]. To address the specific need for biomarker-guided therapy in the elderly, a recent meta-analysis of individual patient data from 28 randomized-controlled trials (RCT) assessed the efficacy and safety of using PCT to guide antibiotic decisions in patients with different types and severities of sepsis stratified in four different age groups (< 75 years [*n* = 7079], 75–80 years [*n* = 1034], 81–85 years [*n* = 803] and > 85 years [*n* = 505]) [30]. This analysis found a reduction in the use of antibiotics between 22 and 26% among all age groups when antibiotics where guided according to a PCT algorithm, with no increase in mortality or adverse outcome risks, showing that a biomarker strategy to guide antibiotic treatment is feasible in the elderly population despite differences in the immune response with age.

Given the importance of an optimal treatment of infections in the elderly patient population, we aimed within a group of experts to critically review and discuss the current literature regarding PCT and other biomarkers for risk stratification and antibiotic stewardship in elderly patients and to find a consensus on different aspects of biomarker use with in a narrative review.

## The aging patient—why is the elderly patient different from the middle-aged or younger patient?

The aging process is characterized by great heterogeneity: indeed, older individuals of the same chronological age may be completely different in functional, cognitive, biological status, responsiveness to stressors (such as an acute infectious disease) and, in the end, health status, length and quality of life. In other terms, while some individuals appear active, fit, healthy and resilient towards stressful events, others are more prone to disease and disabilities, particularly to frailty [31]. Recent studies suggest that a different speed and/or severity of the biological mechanisms of aging, both at cellular level (i.e., mitochondrial and DNA dysfunctions, increased oxidative stress, stem cell exhaustion) and at systemic level (i.e., hormonal dysregulation, chronic inflammatory state, immune system decline or immunosenescence, changes in body composition and neurodegeneration) could explain the phenotypic heterogeneity of elderly people [32]. Indeed, the presence of frailty, independently from multimorbidity and chronological age, may explain the atypical presentation, signs and symptoms of many clinical disorders, including infectious diseases, in the older patients [33]. This is important when assessing the risk of infection in an elderly patient.

Frailty is a multidimensional condition, with physical, functional, psycho-cognitive and socio-economic factors playing a part in its development and evolution [31]. Thus a complete evaluation of frailty to support its management requires a multidimensional approach based on the Comprehensive Geriatric Assessment (CGA) [34], which is today considered the ‘gold standard’ in clinical practice [35]. Currently, very few tools to measure frailty show a multidimensional CGA-based construct able to identify those impaired domains that require a personalized management [36]. The Multidimensional Prognostic Index (MPI) is a CGA-based frailty instrument, extensively validated and implemented among more than 56,000 older adults in different settings (e.g., hospital, nursing home, general practice, community) [37] and with different clinical conditions, including infectious diseases [38, 39]. The MPI gives a clinimetric measure of multidimensional frailty [38] with excellent prognostic value [35, 40, 41]. It also provides useful information in clinical decision making of older patients, for instance in those with acute respiratory failure [42] and SARS-CoV-2 infection. Different versions of the MPI, including the self-reported-MPI [43] and the brief-MPI [44] support its use as screening tool, both in primary care and other non-geriatric settings. Interestingly, the predictive value of MPI can be increased by integrating information of specific biomarkers, such as PCT. Combination of PCT at hospital admission with the MPI significantly increased accuracy of mortality prediction in older patients with community-acquired pneumonia [45].

## Why be extra careful in the elderly population?

Antibiotic overuse in elderly patients may lead to adverse events, including the risk of drug interactions, side effects, risk of *Clostridioides difficile* infections (CDI) and selection of multidrug-resistant (MDR) organisms. In a study focusing on outpatient antibiotic prescribing among older adults in the United States from 2011 to 2014, it has been demonstrated that subjects aged ≥ 75 years had a higher prescribing rate compared to younger persons [46]. In addition, elderly patients may be more prone to side-effects of antibiotics compared to younger patients due to several factors. First, data suggest that the type of antibiotic drug is important as some drugs are more strongly associated with increased risk of adverse events in the elderly compared to younger adults. Altered pharmacokinetics, changes in protein binding and reduced renal function may be the underlying reason for higher rates of adverse events in elderly patients [47]. Compared to younger patients, elderly may be more susceptible to adverse events, especially when some antibiotics, representing the last resort of MDRO infections, are used. There are particular concerns in the use of some antibiotics, such as vancomycin and colistin in elderly patients. Although new antibiotics have been developed for the treatment of methicillin-resistant *Staphylococcus aureus* infections and MDR Gram negative bacilli infections, vancomycin and colistin continue to represent therapeutic options in these patients despite their association with nephrotoxicity. In a study comparing vancomycin use in younger versus older patients, the risk of nephrotoxicity almost tripled in the elderly (7.8 vs. 18.9%, *p* = 0.003) [48]. Similar findings are available for colistin. A recent metanalysis including 2857 elderly with MDRO infections showed that acute kidney injury was significantly more common in patients who received colistin versus other drugs [49]. In addition, not only the type of antibiotics, but also the duration of antibiotic therapy plays an important role in the outcome of elderly patients. The increase in antibiotic-free days has been associated with a reduction in the risk of side effects [50], and for many types of infections there is evidence that shorter treatment courses are equally effective compared to longer ones. A Cochrane review showed that in elderly patients with urinary tract infections (UTI) there was no difference of clinical cure between short course (3–6 days) versus longer antibiotic courses (7–14 days) [51]. Still, in clinical practice, overtreatment of elderly patients is still a major concern, which may again be due to difficulties in understanding clinical signs in this population. In this context, the use of biomarkers may offer a significant and particularly promising opportunity to improve individualized antibiotic therapy and shorten duration of treatment. Finally, when treating elderly patients with infection, the focus should be on patient-centered outcomes. Because older adults are more likely to have complex health care needs [52]. Important goals in this patient population include quality of life and time at home (vs. time spent in the hospital or being institutionalized). Thus, it is important to take a multifaceted approach for guiding antibiotic decisions including parameters such as improvement of clinical conditions, reduction in markers of inflammation and infection, ability to take oral drugs, and patients’ compliance.

## What is procalcitonin and how can it be measured?

Procalcitonin (PCT) is the precursor peptide—or prohormone—of the mature hormone calcitonin [53, 54]. The dual function of PCT as a calcitonin precursor peptide as well as a cytokine mediator, which is elevated upon systemic bacterial infections in line with other cytokines, has resulted in the term “hormokine” mediator [54]. PCT is released ubiquitously by parenchymal cells in response to microbial toxins and to certain cytokines, namely interleukin (IL)-1b, tumor-necrosis factor (TNF)-a and IL-6, among others [54]. Conversely, PCT production is attenuated by certain cytokines released in response to a viral infection, particularly interferon-γ [55]. Although the exact downstream effects of PCT are not entirely clear, there is evidence from preclinical studies demonstrating that PCT plays a pathophysiologic role in the development of severe sepsis and sepsis-related mortality. Administration of PCT to septic hamsters with peritonitis doubled their death rate, while treatment with PCT-reactive antiserum increased survival of septic hamsters and pigs with mono- and polymicrobial sepsis [56–59]. Because PCT is upregulated in bacterial but not viral infections [54, 55], different studies have evaluated the potential of PCT to distinguish viral from bacterial infections as discussed below. Older diagnostic studies used the B.R.A.H.M.S. LUMI test to measure PCT levels [60]. This test detects only markedly elevated PCT levels with a luminometer with a functional assays sensitivity of 0.3–0.5 µg/L. While this test has low sensitivity, currently most physicians use highly sensitive PCT assay with a lower detection limit of 0.06 µg/L such as the BRAHMS Kryptor assay, which is based on a sheep polyclonal anti-calcitonin antibody [61]. Recently, other high sensitive quantitative automated options for PCT testing have become available with functional detection limits in the range of 0.06 µg/L, including the VIDAS system (Biomerieux) [62], the Liaison BRAHMS PCT (DiaSorin) [63], the Elecsys BRAHMS PCT (Roche Diagnostics) [64], the Lumipulse(Fujirebio) [65], the Abbott Architect [66] and the Siemens Advia [67] among others.

## The biomarker approach in the elderly patient

It is important to understand that host biomarkers of sepsis have significantly evolved during the last decades. Biomarkers represent objective indicators of a patient’s clinical condition that can be measured accurately and reproducibly [68]. However, many biomarkers that are routinely used in sepsis may show a different performance in elderly patients due to several factors including the presence of chronic inflammation in this population. For example, patients with frailty are characterized by having lower lymphocyte counts, and higher levels of inflammatory markers including interleukin 6, C-reactive protein (CRP), and tumor necrosis factor-α [69]. In addition, there are also differences in pathogens in the elderly population with an increased rate of multi-resistant organisms due to the greater use of healthcare facilities and cumulative antibiotic exposure [70].

The clinical presentation of sepsis in older patients may be significantly different from younger ones as a result of several factors including differences in cytokine production and more severe infection due to diagnostic delays and higher susceptibility to deterioration [71]. In fact, elderly patients often present with ambiguous and unspecific symptoms, such as altered consciousness, malaise, cognitive impairment, hyporexia, muscle weakness, and incontinence. Importantly, fever as a hallmark of the host response is absent in a significant proportion of elderly patients with infection. Accordingly, prospective studies have shown that commonly used clinical scores for assessing sepsis (e.g., qSOFA, SIRS) have lower sensitivity and specificity in old patients [72]. Elderly patients are also at increased risk for fast deterioration, which significantly correlates with frailty and high burden of comorbidity [73]. The rapid deterioration in the elderly patient adds to the importance of early diagnosis and treatment in this population. High degree of suspicion is crucial to identify patients at risk of clinical worsening to septic shock, multiorgan dysfunction and death. In these time-critical decisions, biomarkers, such as PCT, may improve sepsis management by early detection of elderly patients with severe infections or early sepsis to initiate treatment measures early.

Several studies focusing on elderly patients (> 75 years) have found that similar cut-off values of PCT can be used as in younger patients, particularly when the probability of bacterial infection is high [74]. There are also several studies documenting the clinical benefits of a prompt initiation of appropriate broad-spectrum antimicrobial treatment in elderly patients with sepsis, particularly when multi-drug resistant organisms are suspected [75]. Yet, the decision to stop antibiotic therapy should be based on several parameters, especially in elderly patients with severe infections. Biomarkers may guide clinicians in this choice. However, the identification of the optimal protein biomarker is challenging. Increasing evidence shows that CRP is not only an inflammatory biomarker, but also correlates with ageing-related diseases including cardiovascular disease, hypertension, diabetes mellitus, and kidney disease [76]. As a consequence, many elderly, polymorbid patients have increased levels of inflammatory biomarkers such as IL-6, CRP and TNF-α [77]. Thus, CRP has low specificity in this population. PCT has shown to be more specific and less influenced by polymorbidity [78]. In one study looking at elderly patients, a low PCT cut-off of 0.055 ng/mL had a much higher specificity and sensitivity in predicting bacterial infections compared to CRP (i.e., 92.4% and 63.6% vs., 80.0% and 81.3%, respectively). There are also some interesting findings in elderly patients with COVID-19. In a large observational study, Moreno-García and colleagues found that patients with COVID-19 and higher PCT values had more risk to be affected by bacterial coinfections than those with lower PCT levels [79].

## Treatment studies in respiratory tract infection

While several studies have investigated the value of PCT in elderly patients, today most evidence is available for respiratory tract infection. Herein, PCT has shown high accuracy to predict bacterial versus viral infection, and detect bacterial superinfection in patients with viral pneumonia when PCT values were > 0.10–0.25 ng/ml [80–82]. Also, PCT was found to be helpful in discriminating between atypical bacteria (*Mycoplasma pneumoniae* or *Chlamydophila pneumoniae*) and typical bacteria [80, 81]. When PCT levels were > 0.5 ng/ml the likelihood for *Streptococcus pneumoniae* was very high [83, 84]. High PCT also predicted poor outcome and risk of severe sepsis and septic shock [84–86], and bacteremia [87–90] in several studies. PCT has demonstrated a high negative predictive values of > 94% and specificities > 90%, particularly when low cut-off levels of 0.1–0.25 ng/ml were used [87, 88]. Also, PCT was helpful for clinical and antimicrobial treatment monitoring with a reduction in PCT levels suggesting resolution of illness [82].

Importantly, different studies have shown that the performance of PCT in elderly patients is similar to younger patients in regard to its diagnostic and prognostic properties. The very recent PROPAGE study evaluated the interest of a strategy using serial measurements of PCT to reduce the duration of antibiotic therapy in old patients (≥ 80 years) with pneumonia [91]. In this tudy, antibiotic therapy exposure was reduced in the PCT group as compared to the Control group from a median of 8–10 days with no significant difference in recovery rate. Also, several studies indicated that PCT was superior to other markers regarding diagnosis, prediction of bacteremia and prediction of mortality [92–95]. A meta-analysis including four studies showed that the area under the curve (AUC) was 0.89 (95%CI 0.86–0.92) for identifying systemic bacterial infections in elderly patients [96]. Looking at bacteraemia, comparisons favored PCT over CRP for its NPV of 95% in older populations at a cut-off of 0.5 ng/mL [97], confirming results of an earlier study with sensitivity for PCT of > 90% and an NPV up to 95% for cut-off levels of 0.4–0.5 ng/mL [98].

Many parameters of the mortality risk scores are related to host inflammatory response to infection. The physiological changes related to ageing, comorbidity, polypharmacy and geriatric syndromes in older patients might inhibit adequate response to infection, thereby reducing the prognostic prediction capacity of these scores in these patients [99–101]. Nevertheless, the scores currently available have not been specifically validated in older patients. The studies provide evidence regarding the limitations of the SIRS criteria, qSOFA, and NEWS2 score in identifying high-risk older patients with acute infection, showing an AUC of 0.69 (95% CI 0.61–0.76; *p* < 0.001) for the qSOFA score, 0.65 (95% CI 0.59–0.72; *p* < 0.001) for SIRS, and 0.70 (0.64–0.76) for NEWS2 to predict short-time death [99, 102].

**Fig. 1:**
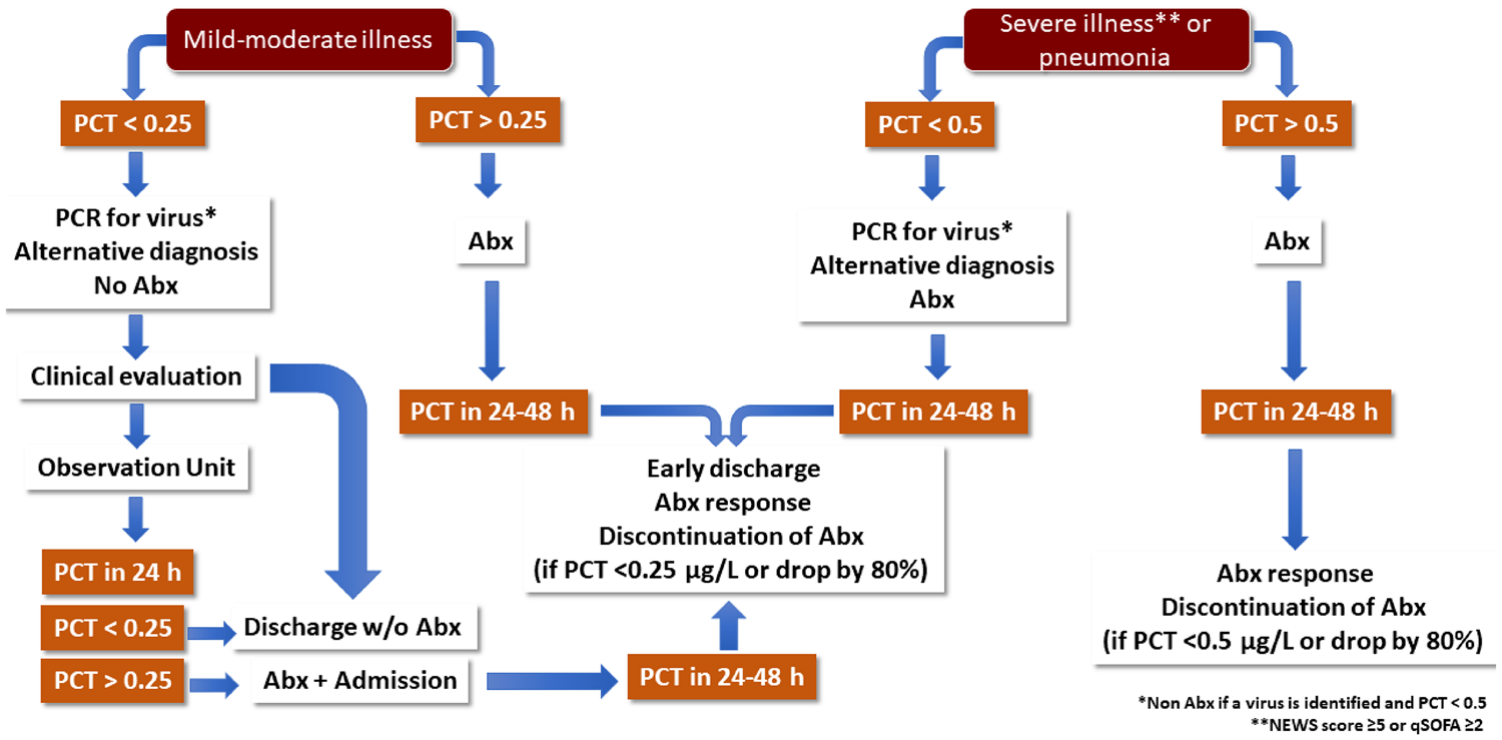
Proposed PCT Algorithm for use in elderly patients mild or severe respiratory infection

Thus, PCT should be used in combination with clinical scoring systems for decision-making, and must be interpreted in the context of clinical findings. Figure 1 shows a proposed algorithm for PCT use in combination with clinical scores for clinical decision making. Severe illness was defined here based on the qSOFA score of ≥ 2 or a NEWS score ≥ 5. We considered pneumonia as severe illness in the elderly patient because it represents the main cause of death due to infectious disease in Western countries (10–14%) [90, 103, 104], and the reason of most episodes of sepsis and septic shock treated in EDs [105], as well as the most prevalent infectious cause (9%) of admission to the intensive care unit (ICU) [83, 105]. In this respect, elderly patients have a more difficult diagnosis [101], greater clinical severity and higher short and long-term mortality [104, 105]. Importantly, this proposed algorithm is based on clinical experience of the authors, but needs validation in prospective trials.

## Treatment studies in urinary tract infections

Urinary tract infections (UTI) are one of the main infections in older populations [71]. At the same time, because of anatomical and functional modifications of bladder, urinary tract mucosa and local immunity, asymptomatic bacteriuria (ASB) becomes more frequent as patients get older. In patients > 80 years of age, 15–50% have ASB [71, 106], defined as the presence of 1 or more species of bacteria growing in the urine at specified quantitative counts (≥ 105 colony-forming units [CFU]/mL or ≥ 108 CFU/L), irrespective of the presence of pyuria, in the absence of signs or symptoms attributable to UTI. However, there is frequent overdiagnosis and overtreatment of ASB in older patients, because they often present with atypical symptoms, which could be due to infection (e.g., delirium). Studies have estimated that 50% of antibiotic treatments for ASB in elderly patients are not required, but importantly increase the risk for side-effects [71, 106]. There is thus need to improve the diagnostic and therapeutic management of the elderly patient with possible UTI. Herein, several studies have investigated the role of PCT in the context of elderly patients presenting with possible UTI.

First, several observational studies investigated PCT in patients presenting with possible UTI. A retrospective single center study from the United States found a negative predictive value of 91% of PCT at a threshold of < 0.25 ng/ml to exclude UTI [107]. However, the negative predictive value was only 77% in another prospective study looking at 229 hospitalized patients with UTI using a PCT threshold of < 0.18 ng/mL [108]. Similarly, in an observational study looking at 216 Dutch nursing home residents from 13 nursing homes, PCT at a threshold of 0.25 ng/ml had a low sensitivity of 35% with a corresponding specificity of 57.8% to diagnose UTI [109]. However, these observational studies were limited by the diagnostic uncertainty around UTI with lack of a strong consensus “goldstandard” diagnosis [106]. In contrast, studies that have looked at PCT to predict bacteremia at thresholds of 0.25 mg/ml to 0.5 ng/ml reported high negative predictive values around 95% [97]. Still, bacteremia may also not be a reliable goldstandard for UTI.

In addition to the above-mentioned observational research, there was also one randomized trial looking at the impact of a PCT-pyuria-based algorithm to guide antibiotic duration in UTI in 125 elderly patients. In this study the PCT protocol decreased the median duration of antibiotic treatment from 10 to 7 days, with similar clinical outcomes at 90 days of follow-up [110]. This pilot study thus suggest that PCT-guided antibiotic treatment reduces antibiotics in the elderly population—but proof of clinical safety has not been well established.

Thus, today, reliably differentiating UTI from ASB in the elderly patients remains an unmet challenge and a goldstandard is largely lacking. There is also only limited evidence demonstrating that PCT would be helpful as a diagnostic tool to limit overtreatment in elderly with observational studies showing mixed results, and one small interventional study providing evidence in favour of PCT use. For patients with more severe UTI and bacteremia, there is more conclusive evidence that a low PCT is helpful in ruling out bacteremia and for guiding duration of antibiotic treatment [97, 110, 111]. Clearly, there is need for additional research regarding PCT in the patients with possible UTI among different settings including older patients in the hospital or nursing homes to understand the clinical benefits of this approach.

## Limitations

This narrative review has limitations. First, we did not do a systematic review for each type of infection but have selected studies based on a PubMed search and the authors expertise. Our conclusions may thus be too enthusiastic. Second, most of the studies did not blind patients and/or investigators and thus subject to possible bias. Third, we did not in more detail discuss other markers of infection. However, there is a lack of well done and large studies comparing the effect of other infection biomarkers when used in the context of antibiotic stewardship in the elderly. Also, there is lack of real world data specifically in the elderly population.

Importantly, PCT is far from being perfect and thus levels must be evaluated in the context of a careful clinical and microbiological patient assessment. Because the kinetics of PCT are of particular diagnostic and prognostic importance, repeated measurements should be performed. This is particularly true for persistently sick patients and in situations where antibiotics are withheld. Limitations of PCT include false-positive and false-negative results [112]. PCT levels may increase in the absence of a bacterial infections in patients with severe trauma or surgery [112–114]. Here, PCT usually shows a rapid decline in follow-up measurements when the patient recovers. Also, chronic renal failure patients may have a slower PCT decrease [115]. PCT levels may also be low in the early course or localised state of an infection with later measurements showing an increase in levels. Thus again, repeated PCT measurements are advised in case of uncertainty. Further, there is lack of interventional research for patients with SARS-CoV-2 infection and this use of PCT in this population remains unproven. Another important consideration are costs of PCT. While some reviews found PCT to be cost efficient in respiratory infections when antibiotics can be reduced by the measurement of this marker [116], this may not be true for other indications. For these specific questions, there is a lack of studies focusing on elderly patients. Also, there is need for studies comparing PCT to more traditional markers such as CRP and ESR. Finally, today, there is no strong guideline recommendation regarding use of PCT in the elderly. This would strengthen the use of this marker in real world practice.

## Future directions and conclusion

This narrative review found several interesting clinical settings where PCT-guided therapy may help to reduce antibiotic exposure by either decreasing initiation or duration of treatment in the elderly population. Yet, PCT and other biomarkers have established roles for diagnosis and prognostication in various diseases albeit ‘patient-centered benefit’ has not been consistently shown, e.g., regarding use of natriuretic peptides to guide therapy of congestive heart failure, a disease associated with advanced age and a classical “domaine” of natriuretic peptides. However, patients in GUIDE-IT received similar care and had similar NT-proBNP lowering regardless of treatment allocation [117]. In this trial, added value of a biomarker-guided approach over ‘carefully managed’ patients could not be documented. Vice versa, the assumption that data regarding management of life-threatening infection can be extrapolated to the elderly is oversimplifying the problem. Regarding the latter, Jung et al. studied the response of elderly patients to corticosteroids for severe COVID19, i.e., a population that is more prone to secondary bacterial infections and other side effects such as hyperglycaemia [118]. They observed a shift in the risk–benefit balance in an elderly, potentially frail patient group consisting of 2115 patients receiving corticosteroids, and 967 patients receiving none. In contrast to the large platform trials including all age groups, 30-day mortality in the elderly was 53% if treated with corticosteroids and 42% in the no-corticosteroid group (*p* < 0.001). This association was even more pronounced after 3 months (69 vs 49%; *p* < 0.001) and was still obvious after multivariable adjustment (aOR 1.60, 95% CI 1.26–2.04; *p* < 0.001). Thus, identification of real-world settings in which elderly patients at risk will favourably respond to a given intervention are required to identify those patients where solely clinical decision making is not sufficient to guide therapy. Today there is an important lack of such real world data in the elderly regarding use of PCT. Still, in the light of the documented potential of PCT to reduce the time on antibiotics in severe infections, use of PCT to guide antibiotics in the elderly is a particularly promising avenue as this population has an altered host response due to immune senescence [30]. The likelihood to develop severe infection along with a blunted clinical appearance remains a leading problem with associated morbidity and mortality warranting to study the added value of biomarkers to support antibiotic stewardship and immunomodulatory interventions, such as corticosteroids.

## Data Availability

There are no original data available for this narrative review.

## References

[1] Shehabi Y, Sterba M, Garrett PM (2014). Procalcitonin algorithm in critically ill adults with undifferentiated infection or suspected sepsis. A randomized controlled trial. Multicenter Study Randomized Controlled Trial Research Support, Non-U.S. Gov’t. Am J Respir Critical Care Med.

[2] Rhodes A, Evans LE, Alhazzani W (2017). Surviving sepsis campaign: international guidelines for management of sepsis and septic shock: 2016. Intensive Care Med.

[3] Kumar A, Ellis P, Arabi Y (2009). Initiation of inappropriate antimicrobial therapy results in a fivefold reduction of survival in human septic shock. Research support non-U.S. Gov't. Chest.

[4] Muller B, Harbarth S, Stolz D (2007). Diagnostic and prognostic accuracy of clinical and laboratory parameters in community-acquired pneumonia. BMC Infect Dis.

[5] Gregoriano C, Heilmann E, Molitor A (2020). Role of procalcitonin use in the management of sepsis. J Thorac Dis.

[6] Neeser O, Branche A, Mueller B (2019). How to: implement procalcitonin testing in my practice. Clin Microbiol Infect.

[7] Branche A, Neeser O, Mueller B (2019). Procalcitonin to guide antibiotic decision making. Curr Opin Infect Dis.

[8] Schuetz P, Birkhahn R, Sherwin R (2017). Serial procalcitonin predicts mortality in severe sepsis patients: results from the multicenter procalcitonin MOnitoring SEpsis (MOSES) Study. Crit Care Med.

[9] Wacker C, Prkno A, Brunkhorst FM (2013). Procalcitonin as a diagnostic marker for sepsis: a systematic review and meta-analysis. Meta-analysis research support non-U.S. Gov’t review. Lancet Infect Dis.

[10] Schuetz P, Kutz A, Grolimund E (2014). Excluding infection through procalcitonin testing improves outcomes of congestive heart failure patients presenting with acute respiratory symptoms: results from the randomized ProHOSP trial. Int J Cardiol.

[11] Mitsuma SF, Mansour MK, Dekker JP (2013). Promising new assays and technologies for the diagnosis and management of infectious diseases. Clin Infect Dis.

[12] Annane D, Maxime V, Faller JP (2013). Procalcitonin levels to guide antibiotic therapy in adults with non-microbiologically proven apparent severe sepsis: a randomised controlled trial. BMJ Open.

[13] Bloos F, Trips E, Nierhaus A (2016). Effect of sodium selenite administration and procalcitonin-guided therapy on mortality in patients with severe sepsis or septic shock: a randomized clinical trial. JAMA Intern Med.

[14] Bouadma L, Luyt CE, Tubach F (2010). Use of procalcitonin to reduce patients' exposure to antibiotics in intensive care units (PRORATA trial): a multicentre randomised controlled trial. Multicenter Study Randomized Controlled Trial Research Support Non-U.S. Gov't. Lancet.

[15] de Jong E, van Oers JA, Beishuizen A (2016). Efficacy and safety of procalcitonin guidance in reducing the duration of antibiotic treatment in critically ill patients: a randomised, controlled, open-label trial. Lancet Infect Dis.

[16] Deliberato RO, Marra AR, Sanches PR (2013). Clinical and economic impact of procalcitonin to shorten antimicrobial therapy in septic patients with proven bacterial infection in an intensive care setting Randomized Controlled Trial. Diagnostic Microbiol Infect Dis.

[17] Hochreiter M, Schroeder S (2011) [Procalcitonin-based algorithm. Management of antibiotic therapy in critically ill patients]. Review. Anaesthesist 60:661–73. Prokalzitoninbasierte Algorithmen. Steuerung der Antibiotikatherapie bei kritisch kranken Patienten. 10.1007/s00101-011-1884-1 10.1007/s00101-011-1884-1 21660525

[18] Schroeder S, Hochreiter M, Koehler T (2009). Procalcitonin (PCT)-guided algorithm reduces length of antibiotic treatment in surgical intensive care patients with severe sepsis: results of a prospective randomized study. Langenbecks Arch Surg.

[19] Hochreiter M, Kohler T, Schweiger AM (2009). Procalcitonin to guide duration of antibiotic therapy in intensive care patients: a randomized prospective controlled trial. Crit Care.

[20] Layios N, Lambermont B, Canivet JL (2012). Procalcitonin usefulness for the initiation of antibiotic treatment in intensive care unit patients. Randomized Controlled Trial. Crit Care Med.

[21] Oliveira CF, Botoni FA, Oliveira CR (2013). Procalcitonin versus C-reactive protein for guiding antibiotic therapy in sepsis: a randomized trial. Randomized controlled trial research support non-U.S. Gov’t. Crit Care Med.

[22] Nobre V, Harbarth S, Graf JD (2008). Use of procalcitonin to shorten antibiotic treatment duration in septic patients: a randomized trial. Am J Respir Crit Care Med.

[23] Wirz Y, Meier MA, Bouadma L (2018). Effect of procalcitonin-guided antibiotic treatment on clinical outcomes in intensive care unit patients with infection and sepsis patients: a patient-level meta-analysis of randomized trials. Crit Care.

[24] Schuetz P, Wirz Y, Sager R (2017). Procalcitonin to initiate or discontinue antibiotics in acute respiratory tract infections. Cochrane Database Syst Rev.

[25] Schuetz P, Briel M, Christ-Crain M (2012). Procalcitonin to guide initiation and duration of antibiotic treatment in acute respiratory infections: an individual patient data meta-analysis. Clin Infect Dis.

[26] Schuetz P, Christ-Crain M, Thomann R (2009). Effect of procalcitonin-based guidelines vs standard guidelines on antibiotic use in lower respiratory tract infections: the ProHOSP randomized controlled trial. JAMA.

[27] Schuetz P, Briel M, Christ-Crain M (2008). Procalcitonin to initiate or withhold antibiotics in acute respiratory tract infections. Cochrane Database Systematic Rev.

[28] Fuentes E, Fuentes M, Alarcon M (2017). Immune system dysfunction in the elderly. An Acad Bras Cienc.

[29] Ticinesi A, Lauretani F, Nouvenne A (2017). C-reactive protein (CRP) measurement in geriatric patients hospitalized for acute infection. Eur J Intern Med.

[30] Heilmann E, Gregoriano C, Annane D (2021). Duration of antibiotic treatment using procalcitonin-guided treatment algorithms in older patients: a patient-level meta-analysis from randomized controlled trials. Age Ageing.

[31] Hoogendijk EO, Afilalo J, Ensrud KE (2019). Frailty: implications for clinical practice and public health. Lancet.

[32] Zampino M, Polidori MC, Ferrucci L (2022). Biomarkers of aging in real life: three questions on aging and the comprehensive geriatric assessment. Geroscience.

[33] Poco PCE, Aliberti MJR, Dias MB (2021). Divergent: age, frailty, and atypical presentations of COVID-19 in hospitalized patients. J Gerontol A Biol Sci Med Sci.

[34] Turner G, Clegg A, British Geriatrics S, Age UK, College Royal, of General P (2014). Best practice guidelines for the management of frailty: a British Geriatrics Society, age UK and Royal College of General Practitioners report. Age Ageing.

[35] Wahlich J, Orlu M, Mair A (2019). Age-related medicine. Pharmaceutics.

[36] Dent E, Martin FC, Bergman H (2019). Management of frailty: opportunities, challenges, and future directions. Lancet.

[37] Veronese N, Custodero C, Cella A (2021). Prevalence of multidimensional frailty and pre-frailty in older people in different settings: a systematic review and meta-analysis. Ageing Res Rev.

[38] Pilotto A, Custodero C, Maggi S (2020). A multidimensional approach to frailty in older people. Ageing Res Rev.

[39] Verholt AB, Gregersen M, Gonzalez-Bofill N (2021). Clinical presentation and outcomes of COVID-19 in older hospitalised patients assessed by the record-based multidimensional prognostic index, a cross-sectional study. Eur Geriatr Med.

[40] Mattace-Raso F, Pilotto A (2021). The challenge of the multifaceted prognosis in the older people and the Multidimensional Prognostic Index. Eur Geriatr Med.

[41] Cruz-Jentoft AJ, Daragjati J, Fratiglioni L (2020). Using the Multidimensional Prognostic Index (MPI) to improve cost-effectiveness of interventions in multimorbid frail older persons: results and final recommendations from the MPI_AGE European Project. Aging Clin Exp Res.

[42] Custodero C, Gandolfo F, Cella A (2021). Multidimensional prognostic index (MPI) predicts non-invasive ventilation failure in older adults with acute respiratory failure. Arch Gerontol Geriatr.

[43] Zora S, Custodero C, Pers YM (2021). Impact of the chronic disease self-management program (CDSMP) on self-perceived frailty condition: the EU-EFFICHRONIC project. Ther Adv Chronic Dis.

[44] Cella A, Veronese N, Custodero C (2022). Validation of abbreviated form of the multidimensional prognostic index (MPI): the BRIEF-MPI project. Clin Interv Aging.

[45] Pilotto A, Dini S, Daragjati J (2018). Combined use of the multidimensional prognostic index (MPI) and procalcitonin serum levels in predicting 1-month mortality risk in older patients hospitalized with community-acquired pneumonia (CAP): a prospective study. Aging Clin Exp Res.

[46] Kabbani S, Palms D, Bartoces M (2018). Outpatient antibiotic prescribing for older adults in the United States: 2011 to 2014. J Am Geriatr Soc.

[47] Falcone M, Paul M, Tiseo G (2020). Considerations for the optimal management of antibiotic therapy in elderly patients. J Glob Antimicrob Resist.

[48] Vance-Bryan K, Rotschafer JC, Gilliland SS (1994). A comparative assessment of vancomycin-associated nephrotoxicity in the young versus the elderly hospitalized patient. J Antimicrob Chemother.

[49] Margalit I, Prendki V, Tishler O (2022). Effectiveness and safety of colistin among older adults: a systematic review and meta-analysis. J Antimicrob Chemother.

[50] Tamma PD, Avdic E, Li DX (2017). Association of adverse events with antibiotic use in hospitalized patients. JAMA Intern Med.

[51] Lutters M, Vogt N (2002). Antibiotic duration for treating uncomplicated, symptomatic lower urinary tract infections in elderly women. Cochrane Database Syst Rev.

[52] Jayadevappa R (2017). Patient-centered outcomes research and patient-centered care for older adults: a perspective. Gerontol Geriatr Med.

[53] Becker KL, Nylen ES, White JC (2004). Clinical review 167: procalcitonin and the calcitonin gene family of peptides in inflammation, infection, and sepsis: a journey from calcitonin back to its precursors. J Clin Endocrinol Metab.

[54] Muller B, Becker KL (2001). Procalcitonin: how a hormone became a marker and mediator of sepsis. Swiss Med Wkly.

[55] Linscheid P, Seboek D, Nylen ES (2003). In vitro and in vivo calcitonin I gene expression in parenchymal cells: a novel product of human adipose tissue. Endocrinology.

[56] Becker KL, Nylen ES, Snider RH (2003). Immunoneutralization of procalcitonin as therapy of sepsis. J Endotoxin Res.

[57] Wagner KE, Martinez JM, Vath SD (2002). Early immunoneutralization of calcitonin precursors attenuates the adverse physiologic response to sepsis in pigs. Crit Care Med.

[58] Martinez JM, Wagner KE, Snider RH (2001). Late immunoneutralization of procalcitonin arrests the progression of lethal porcine sepsis. Surg Infect (Larchmt).

[59] Nylen ES, Whang KT, Snider RH (1998). Mortality is increased by procalcitonin and decreased by an antiserum reactive to procalcitonin in experimental sepsis. Crit Care Med.

[60] Schuetz P, Bretscher C, Bernasconi L (2017). Overview of procalcitonin assays and procalcitonin-guided protocols for the management of patients with infections and sepsis. Expert Rev Mol Diagn.

[61] Steinbach G, Rau B, Debard AL (2004). Multicenter evaluation of a new immunoassay for procalcitonin measurement on the Kryptor System. Clin Chem Lab Med.

[62] Schuetz P, Christ-Crain M, Huber AR (2009). Long-term stability of procalcitonin in frozen samples and comparison of Kryptor(R) and VIDAS(R) automated immunoassays. Clin Biochem.

[63] Hubl W, Krassler J, Zingler C (2003). Evaluation of a fully automated procalcitonin chemiluminescence immunoassay. Clin Lab.

[64] de Wolf HK, Gunnewiek JK, Berk Y (2009). Comparison of a new procalcitonin assay from roche with the established method on the brahms kryptor. Clin Chem.

[65] Dupuy AM, Ne M, Bargnoux AS (2017). Analytical evaluation of Lumipulse(R) BRAHMS PCT CLEIA assay and clinical performances in an unselected population as compared with central lab PCT assay. Clin Biochem.

[66] Soh A, Binder L, Clough M (2018). Comparison of the novel ARCHITECT procalcitonin assay with established procalcitonin assay systems. Pract Lab Med.

[67] Sanders RJ, Schoorl M, Dekker E (2011). Evaluation of a new procalcitonin assay for the Siemens ADVIA Centaur with the established method on the BRAHMS Kryptor. Clin Lab.

[68] Barichello T, Generoso JS, Singer M (2022). Biomarkers for sepsis: more than just fever and leukocytosis-a narrative review. Crit Care.

[69] Xu Y, Wang M, Chen D (2022). Inflammatory biomarkers in older adults with frailty: a systematic review and meta-analysis of cross-sectional studies. Aging Clin Exp Res.

[70] Kirby JT, Fritsche TR, Jones RN (2006). Influence of patient age on the frequency of occurrence and antimicrobial resistance patterns of isolates from hematology/oncology patients: report from the Chemotherapy Alliance for Neutropenics and the Control of Emerging Resistance Program (North America). Diagn Microbiol Infect Dis.

[71] Gavazzi G, Krause KH (2002). Ageing and infection. Lancet Infect Dis.

[72] Bastoni D, Ticinesi A, Lauretani F (2019). Application of the sepsis-3 consensus criteria in a geriatric acute care unit: a prospective study. J Clin Med.

[73] Mensa J, Barberán J, Ferrer R (2021). Recommendations for antibiotic selection for severe nosocomial infections. Rev Esp Quimioter.

[74] Gómez-Cerquera JM, Daroca-Pérez R, Baeza-Trinidad R (2015). Validity of procalcitonin for the diagnosis of bacterial infection in elderly patients. Enferm Infecc Microbiol Clin.

[75] Zilberberg MD, Shorr AF, Micek ST (2014). Multi-drug resistance, inappropriate initial antibiotic therapy and mortality in Gram-negative severe sepsis and septic shock: a retrospective cohort study. Crit Care.

[76] Tang Y, Fung E, Xu A (2017). C-reactive protein and ageing. Clin Exp Pharmacol Physiol.

[77] Velissaris D, Pantzaris N, Koniari I (2017). C-reactive protein and frailty in the elderly: a literature review. J Clin Med Res.

[78] Yan L, Liao P, Xu LL (2014). Usefulness of procalcitonin in elderly patients with bacterial infection. Clin Lab.

[79] Moreno-Garcia E, Puerta-Alcalde P, Letona L (2022). Bacterial co-infection at hospital admission in patients with COVID-19. Int J Infect Dis.

[80] Alba GA, Truong QA, Gaggin HK, et al (2016) Diagnostic and prognostic utility of procalcitonin in patients presenting to the emergency department with dyspnea. Am J Med 129:96–104e7. 10.1016/j.amjmed.2015.06.037 10.1016/j.amjmed.2015.06.037 26169892

[81] Espana PP, Capelastegui A, Bilbao A (2012). Utility of two biomarkers for directing care among patients with non-severe community-acquired pneumonia. Eur J Clin Microbiol Infect Dis.

[82] Julian-Jimenez A, Candel-Gonzalez FJ, et al (2014) [Usefulness of inflammation and infection biomarkers in the Emergency Department]. Enferm Infecc Microbiol Clin 32:177–90. Utilidad de los biomarcadores de inflamacion e infeccion en los servicios de urgencias. 10.1016/j.eimc.2013.01.005 10.1016/j.eimc.2013.01.005 23490142

[83] Monclus Cols E, Capdevila Reniu A, Roedberg Ramos D, et al (2016) [Management of severe sepsis and septic shock in a tertiary care urban hospital emergency department: opportunities for improvement]. Emergencias. Ago 28:229–234. Manejo de la sepsis grave y el shock septico en un servicio de urgencias de un hospital urbano de tercer nivel. Oportunidades de mejora 29105408

[84] Vail GM, Xie YJ, Haney DJ (2009). Biomarkers of thrombosis, fibrinolysis, and inflammation in patients with severe sepsis due to community-acquired pneumonia with and without *Streptococcus pneumoniae*. Infection.

[85] Julian-Jimenez A, Timon Zapata J, Laserna Mendieta EJ, et al (2014) [Diagnostic and prognostic power of biomarkers to improve the management of community acquired pneumonia in the emergency department]. Enferm Infecc Microbiol Clin 32:225–235. Poder diagnostico y pronostico de los biomarcadores para mejorar el manejo de la neumonia adquirida en la comunidad en los servicios de urgencias. 10.1016/j.eimc.2013.04.015 10.1016/j.eimc.2013.04.015 24182623

[86] Viasus D, Del Rio-Pertuz G, Simonetti AF (2016). Biomarkers for predicting short-term mortality in community-acquired pneumonia: a systematic review and meta-analysis. J Infect.

[87] Muller F, Christ-Crain M, Bregenzer T (2010). Procalcitonin levels predict bacteremia in patients with community-acquired pneumonia: a prospective cohort trial. Chest.

[88] Julian-Jimenez A, Timon Zapata J, Laserna Mendieta EJ, et al (2014) [Ability of procalcitonin to predict bacteremia in patients with community acquired pneumonia]. Med Clin (Barc). 142:285–92. Capacidad de la procalcitonina para predecir bacteriemia en pacientes con neumonia adquirida en la comunidad. 10.1016/j.medcli.2013.05.046 10.1016/j.medcli.2013.05.046 24120103

[89] Rubio-Diaz R, Julian-Jimenez A, Gonzalez Del Castillo J, et al (2022) Ability of lactate, procalcitonin, and criteria defining sepsis to predict 30 day mortality, bacteremia, and microbiologically confirmed infection in patients with infection suspicion treated in emergency departments. Emergencias 34:181–189. Capacidad del lactato, procalcitonina y de los criterios definitorios de sepsis para predecir mortalidad a 30 dias, bacteriemia o infeccion confirmada microbiologicamente en los pacientes atendidos por sospecha de infeccion en urgencias 35736522

[90] Julian-Jimenez A, Gonzalez Del Castillo J, Candel FJ (2017) Usefulness and prognostic value of biomarkers in patients with community-acquired pneumonia in the emergency department. Med Clin (Barc) 148:501–510. Utilidad y valor pronostico de los biomarcadores en los pacientes con neumonia adquirida en la comunidad en los servicios de urgencias. 10.1016/j.medcli.2017.02.024 10.1016/j.medcli.2017.02.024 28391994

[91] Gavazzi G, Drevet S, Debray M (2022). Procalcitonin to reduce exposure to antibiotics and individualise treatment in hospitalised old patients with pneumonia: a randomised study. BMC Geriatrics.

[92] Poole S, Kidd SP, Saeed K (2018). A review of novel technologies and techniques associated with identification of bloodstream infection etiologies and rapid antimicrobial genotypic and quantitative phenotypic determination. Expert Rev Mol Diagn.

[93] Charles PE, Ladoire S, Snauwaert A (2008). Impact of previous sepsis on the accuracy of procalcitonin for the early diagnosis of blood stream infection in critically ill patients. BMC Infect Dis.

[94] Saeed K, Gonzalez Del Castillo J, Backous C (2019). Hot topics on procalcitonin use in clinical practice, can it help antibiotic stewardship?. Int J Antimicrob Agents.

[95] Gavazzi G, Meyrignac L, Zerhouni N (2022). Intrinsic values of procalcitonin in bacterial bloodstream infections in people aged 75 years and over: a retrospective study. Diagn Microbiol Infect Dis.

[96] Lee SH, Chan RC, Wu JY (2013). Diagnostic value of procalcitonin for bacterial infection in elderly patients—a systemic review and meta-analysis. Int J Clin Pract.

[97] Hoeboer SH, van der Geest PJ, Nieboer D (2015). The diagnostic accuracy of procalcitonin for bacteraemia: a systematic review and meta-analysis. Clin Microbiol Infect.

[98] Lai CC, Chen SY, Wang CY (2010). Diagnostic value of procalcitonin for bacterial infection in elderly patients in the emergency department. J Am Geriatr Soc.

[99] Gonzalez Del Castillo J, Julian-Jimenez A, Gonzalez-Martinez F (2017). Prognostic accuracy of SIRS criteria, qSOFA score and GYM score for 30 day-mortality in older non-severely dependent infected patients attended in the emergency department. Eur J Clin Microbiol Infect Dis.

[100] Martin GS, Mannino DM, Moss M (2006). The effect of age on the development and outcome of adult sepsis. Crit Care Med.

[101] Martin-Sanchez FJ, Gonzalez Del Castillo J (2015) [Sepsis in the elderly: are hospital emergency departments prepared?]. Emergencias 27:73–74. Sepsis en el anciano: inverted question markestan preparados los servicios de urgencias hospitalarios? 29077346

[102] Kemp K, Alakare J, Harjola VP (2020). National early warning score 2 (NEWS2) and 3-level triage scale as risk predictors in frail older adults in the emergency department. BMC Emerg Med.

[103] Mandell LA, Wunderink RG, Anzueto A (2007). Infectious Diseases Society of America/American Thoracic Society consensus guidelines on the management of community-acquired pneumonia in adults. Clin Infect Dis.

[104] Torres A, Barberan J, Falguera M, et al (2013) [Multidisciplinary guidelines for the management of community-acquired pneumonia]. Med Clin (Barc) 140(5):223 e1–223 e19. Guia multidisciplinar para la valoracion pronostica, diagnostico y tratamiento de la neumonia adquirida en la comunidad. 10.1016/j.medcli.2012.09.034 10.1016/j.medcli.2012.09.034 23276610

[105] Julian Jimenez A, Gonzalez del Castillo J, Martinez Ortiz de Zarate M et al (2013) [Characteristics and epidemiological changes for patients with community-acquired pneumonia in hospital emergency departments]. An Sist Sanit Navar. 36:387–95. Caracteristicas y cambios epidemiologicos de los pacientes con neumonia adquirida en la comunidad en los servicios de urgencias hospitalarios. 10.4321/s1137-66272013000300004 10.4321/s1137-66272013000300004 24406352

[106] Nicolle LE, Gupta K, Bradley SF (2019). Clinical practice guideline for the management of asymptomatic bacteriuria: 2019 update by the Infectious Diseases Society of America. Clin Infect Dis.

[107] Levine AR, Tran M, Shepherd J (2018). Utility of initial procalcitonin values to predict urinary tract infection. Am J Emerg Med.

[108] Choi JJ, McCarthy MW, Meltzer KK (2022). The diagnostic accuracy of procalcitonin for urinary tract infection in hospitalized older adults: a prospective study. J Gen Intern Med.

[109] Kuil SD, Hidad S, Fischer JC (2021). Sensitivity of C-reactive protein and procalcitonin measured by point-of-care tests to diagnose urinary tract infections in nursing home residents: a cross-sectional study. Clin Infect Dis.

[110] Drozdov D, Schwarz S, Kutz A (2015). Procalcitonin and pyuria-based algorithm reduces antibiotic use in urinary tract infections: a randomized controlled trial. BMC Med.

[111] Meier MA, Branche A, Neeser OL (2019). Procalcitonin-guided antibiotic treatment in patients with positive blood cultures: a patient-level meta-analysis of randomized trials. Clin Infect Dis.

[112] Christ-Crain M, Muller B (2005). Procalcitonin in bacterial infections–hype, hope, more or less?. Swiss Med Wkly.

[113] Uzzan B, Cohen R, Nicolas P (2006). Procalcitonin as a diagnostic test for sepsis in critically ill adults and after surgery or trauma: a systematic review and meta-analysis. Crit Care Med.

[114] Hunziker S, Hugle T, Schuchardt K (2010). The value of serum procalcitonin level for differentiation of infectious from noninfectious causes of fever after orthopaedic surgery. J Bone Jt Surg.

[115] Heilmann E, Gregoriano C, Wirz Y (2020). Association of kidney function with effectiveness of procalcitonin-guided antibiotic treatment: a patient-level meta-analysis from randomized controlled trials. Clin Chem Lab Med.

[116] Schuetz P, Balk R, Briel M (2015). Economic evaluation of procalcitonin-guided antibiotic therapy in acute respiratory infections: a US health system perspective. Clin Chem Lab Med.

[117] Ibrahim NE, Januzzi JL (2018). The Future of Biomarker-Guided Therapy for Heart Failure After the Guiding Evidence-Based Therapy Using Biomarker Intensified Treatment in Heart Failure (GUIDE-IT) Study. Curr Heart Fail Rep.

[118] Jung C, Wernly B, Fjolner J (2021). Steroid use in elderly critically ill COVID-19 patients. Eur Respir J.

